# The effectiveness of theory-based smoking cessation interventions in patients with chronic obstructive pulmonary disease: a meta-analysis

**DOI:** 10.1186/s12889-023-16441-w

**Published:** 2023-08-09

**Authors:** Mengjing Han, Yingping Fu, Quanyue Ji, Xiaoli Deng, Xuewen Fang

**Affiliations:** 1 Yunnan University of Chinese Medicine, Kunming, Yunnan People’s Republic of China; 2 The First People’s Hospital of Yunnan Province, Kunming, Yunnan People’s Republic of China

**Keywords:** Chronic obstructive pulmonary disease, Smoking cessation, Theory, Clinical trial, Nursing, Meta-analysis

## Abstract

**Background:**

Smoking cessation can effectively reduce the risk of death, alleviate respiratory symptoms, and decrease the frequency of acute exacerbations in patients with chronic obstructive pulmonary disease (COPD). Effective smoking cessation strategies are crucial for the prevention and treatment of COPD. Currently, clinical interventions based on theoretical frameworks are being increasingly used to help patients quit smoking and have shown promising results. However, theory-guided smoking cessation interventions have not been systematically evaluated or meta-analyzed for their effectiveness in COPD patients. To improve smoking cessation rates, this study sought to examine the effects of theory-based smoking cessation interventions on COPD patients.

**Methods:**

We adhered to the PRISMA guidelines for our systematic review and meta-analysis. The Cochrane Library, Web of Science, PubMed, Embase, Wanfang, CNKI, VIP Information Services Platform, and China Biomedical Literature Service System were searched from the establishment of the database to April 20, 2023. The study quality was assessed using the Cochrane Collaboration's risk assessment tool for bias. The revman5.4 software was used for meta-analysis. The *I*^*2*^ test was used for the heterogeneity test, the random effect model and fixed effect model were used for meta-analysis, and sensitivity analysis was performed by excluding individual studies.

**Results:**

A total of 11 RCTs involving 3,830 patients were included in the meta-analysis. Results showed that theory-based smoking cessation interventions improved smoking cessation rates, quality of life, and lung function in COPD patients compared to conventional nursing. However, these interventions did not significantly affect the level of nicotine dependence in patients.

**Conclusion:**

Theory-based smoking cessation intervention as a non-pharmacologically assisted smoking cessation strategy has a positive impact on motivating COPD patients to quit smoking and improving their lung function and quality of life.

**Trial registration:**

PROSPERO registration Number: CRD42023434357.

**Supplementary Information:**

The online version contains supplementary material available at 10.1186/s12889-023-16441-w.

## Introduction

Chronic Obstructive Pulmonary Disease (COPD) is a heterogeneous lung disease characterized by persistent respiratory symptoms and airflow limitation caused by airway and/or alveolar abnormalities, as defined by the 2023 Global Initiative for Chronic Obstructive Lung Disease (GOLD) [1]. In China, the overall prevalence of COPD is 8.6%, with a rate of 13.7% in the population over 40 years old [2]. Smoking is a major risk factor for COPD, with smokers having 10.92 times the risk of developing COPD compared to non-smokers [3]. Additionally, smoking COPD patients have more respiratory symptoms than non-smokers and higher mortality rates [4]. Smoking cessation is considered the most effective and cost-effective strategy for preventing and treating COPD [5]. For COPD smokers, it is important to adopt effective methods to control their smoking behavior [6]. However, smoking cessation is challenging, and conventional approaches may not be effective for all patients. Although conventional smoking cessation methods such as telephone hotlines [7], medication [8], and comprehensive interventions [9] have been shown to improve patients' smoking cessation rates and lung function to some extent, patients' smoking cessation behavior is highly influenced by their health knowledge and behavior change.

Therefore, some scholars have attempted to use theory-guided interventions to improve COPD patients' smoking cessation rates, achieving good results. Currently, the theories related to the management of smoking cessation in COPD include "timing theory" [10], "theory of planned behavior" [11], "the 5A nursing model" [12], and "cognitive-behavioral theory" [13]. The timing theory was proposed by Canadian scholars Cameron et al [10]. According to this theory, targeted intervention should be implemented according to the disease stage of patients, emphasizing the importance of understanding the different stages of the disease, focusing on the patients themselves, increasing their confidence in treating the disease, improving their current negative behaviors and emotions, and ultimately achieving a positive health outcome [14, 15]. The planned behavior theory was proposed by Ajzen [11], who believed that individual behavior is mainly influenced by individual behavioral intentions, including attitudes, subjective norms, and perceived behavioral control. Attitude refers to the positive or negative evaluation and experience of behavior; subjective norms refer to the social pressure felt when adopting behavior; and perceived behavioral control refers to self-efficacy and control over behavior [16, 17]. The 5A nursing model [12] includes five components: assess, advise, agree, assist, and arrange. The aim is to improve patients' self-efficacy and self-management skills [18, 19]. Cognitive-behavioral theory is an integration of cognitive theory and behavioral theory that utilizes methods to change negative cognitions, beliefs, and behaviors [13]. Cognitive-behavioral interventions involve selecting theories related to cognition and/or behavior, considering individual, behavioral, and environmental factors, and designing intervention plans based on the individual's understanding of behavior change and available resources. This approach promotes the formation of healthy behaviors and corrects negative ones [20]. Theory-based smoking cessation interventions are designed to provide patients with the knowledge, skills, and support necessary to quit smoking successfully [21]. By understanding these theories, healthcare providers can design interventions that are tailored to the individual patient's needs and increase the likelihood of successful smoking cessation [22].

Currently, there has yet to be a systematic evaluation or meta-analysis of the effectiveness of theory-based smoking cessation interventions in COPD patients. Therefore, this study aims to synthesize randomized controlled trials of theory-based smoking cessation interventions in COPD patients and evaluate their effectiveness and impact on patients through meta-analysis, providing evidence-based medicine for their clinical application.

## Methods

### Aims

Our aim was to evaluate the effectiveness of theory-based smoking cessation interventions in patients with COPD.

## Design

We followed the Cochrane Collaboration's Preferred Reporting Items for Systematic Reviews and Meta-Analyses (PRISMA) guidelines [23]. The review protocol is registered on the PROSPERO database (Registration No: CRD42023434357).

### Literature search

Two researchers searched for RCT studies published in the Cochrane Library, Web of Science, PubMed, Embase, Wanfang Knowledge Service Platform, CNKI, VIP Resource Integration Service Platform, and China Biomedical Literature Database. The search terms included chronic obstructive pulmonary disease*/chronic obstructive lung disease*/COPD, smoking/smoking cessation/smoking intervention, theory/model/theoretical. We conducted the search by combining subject terms and free words, and expanded our search by tracing the references included in the study in a snowball manner. The retrieval deadline for this search is from the establishment of the database up until April 20, 2023.

### Study selection

The inclusion and exclusion criteria were formulated according to the Population, Intervention, Comparison, Outcome, Study design (PICOS) framework. Inclusion criteria: (i) the study participants met the diagnostic criteria for COPD of the Chinese Medical Association Respiratory Disease Society (2021 revised edition) [24] and also met the relevant criteria for tobacco dependence in the Chinese Clinical Smoking Cessation Guidelines (2015 edition) [25]; (ii) the intervention was based on theoretical smoking cessation methods; (iii) the outcome indicators: at least one of smoking cessation rate, nicotine dependence level, lung function, quality of life, clinical composite symptom score, and number of clinical symptom exacerbations; (iv) the study type was a randomized controlled trial. Exclusion criteria: Exclusion criteria: (i) duplicate publications; (ii) there were no relevant outcome indicators; (iii) literature with incomplete data and outcome index data that cannot be transformed and used; (iv) literature of low quality (based on a Cochrane Collaboration risk of bias assessment quality grade of C).

### Quality assessment

The Cochrane Collaboration's risk of bias assessment tool (RoB 2.0) [26] was used to evaluate the methodological quality of the included studies. Involving seven items: (i) random sequence generation, (ii) allocation concealment, (iii) blinding of participants and personnel, (iv) blinding of outcome assessment, (v) incomplete outcome data (loss to follow-up or withdrawal), (vi) selective reporting, (vii) other biases. High-risk, low-risk, and unclear were used to evaluate the risk of bias for each item. If all of the above criteria are fully met, the study quality level is A, indicating a low possibility of various biases occurring. If some of the above criteria are met, the study quality level is B, indicating a moderate possibility of bias occurring. If none of the above criteria are met, the study quality level is C, indicating a high possibility of bias occurring. In the event of disagreement between the two researchers, a third-party researcher should be consulted to reach a consensus.

### Data extraction

Two researchers independently screened articles, extracted data, and cross-checked them. The data were extracted according to the designed extraction strategy, which included: (i) basic information of the included studies, including title, first author, publication year, abstract, and source of the literature; (ii) study characteristics, including sample size, age of the experimental and control groups, and intervention measures; (iii) outcome indicators, including observation indicators, measurement tools or assessment criteria, measurement values, and research conclusions.

### Data synthesis and analysis

RevMan5.4 software was used for meta-analysis. The heterogeneity test was performed using the *I*^*2*^ test. If P>0.1 and *I*^*2*^ <50%, heterogeneity was considered acceptable, and the fixed effect model was selected; if *P*≤0.1 and *I*^*2*^≥50%, indicated that there was heterogeneity among studies, and the random effect model was selected. A sensitivity analysis was conducted to identify sources of heterogeneity. The effect size of count data was expressed as odds ratio (OR) with a 95% confidence interval (CI), while continuous data were expressed as mean difference (MD) or standardized mean difference (SMD) with a 95% confidence interval (CI).

## Results

### Literature search outcomes

**Fig. 1:**
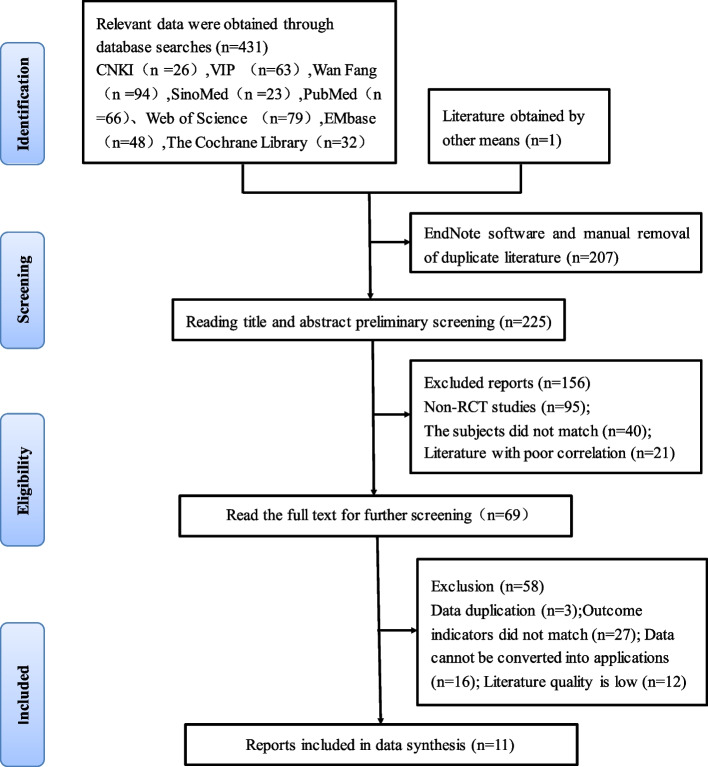
Flow chart of literature screening

We searched 431 relevant articles in the database and obtained one article by reading the references to related studies. The EndNote software was applied to remove 207 duplicate literatures. 156 articles were excluded based on reading the titles and abstracts, as they included non-randomized controlled trials, inconsistent research subjects, and poor correlation. Further reading of the full text was re-screened to exclude 58 papers with the same data, outcome indicators that did not match, data that could not be translated into application, and lower quality. Ultimately, we included 11 articles [27–37] in our analysis, consisting of 9 Chinese-language articles [27–35] and 2 English-language articles [36, 37]. A total of 3830 patients were included, including 1989 in the experimental group and 1841 in the control group. The literature screening process and results are shown in Fig. 1.

### The basic characteristics of studies

11 RCTs published between 2013 and 2023 were included in the meta-analysis. The studies were based on three different theories, including seven on the timing theory [27–33], two on the 5A nursing model [34, 35], and two on the cognitive-behavioral theory [36, 37]. One study on the theory of planned behavior [38] was not included in the meta-analysis because it was not an RCT. The basic characteristics of the literature are shown in Table 1.

**Table 1:** Basic characteristics of the included literature

Inclusion study	Sample	Age		Intervention methods		Outcome indicators
	T/C	T	C	T	C	
Xiang Q et al 2020 [27]	47/49	62.75±11.34	64.52±13.94	the timing theory	routine nursing care	①②
Zhou B et al 2022 [28]	60/60	62.89±2.87	63.10±4.12	the timing theory	routine nursing care	①②③⑤
Shen L et al 2022 [29]	58/55	63.39±9.28	63.08±9.45	the timing theory	routine nursing care	①②③④
Zhang H et al [30]	54/54	53.90±6.31	53.26±6.53	the timing theory	routine nursing care	①②③④
Dang J et al 2022 [31]	61/61	60.42±2.75	60.18±2.69	the timing theory	routine nursing care	①②④
Chen X 2022 [32]	49/49	62.76±11.35	64.49±13.96	the timing theory	routine nursing care	①②③
Yu W et al 2021 [33]	53/53	67.83±9.62	68.25±9.75	the timing theory	routine nursing care	①③⑥
Xu L et al 2018 [34]	79/79	57.53±3.04	55.94 ± 2.67	the 5A nursing model	routine nursing care	⑤⑥
Zhu H et al 2018 [35]	100/100	58.81±11.49	59.73±11.64	the 5A nursing model	routine nursing care	①③④
Lei S et al 2020 [36]	51/51	61.10±12.30	61.50±12.50	the cognitive behavioral theory	routine nursing care	①③
Lou P et al 2013 [37]	1377/1230	61.60±10.20	61.50±10.10	the cognitive behavioral theory	routine nursing care	①②

T: test group, C: control group; ①quit rate; ②nicotine dependence; ③lung function; ④quality of life; ⑤clinical symptom score; ⑥frequency of clinical symptom exacerbation

### Quality assessment

**Fig. 2:**
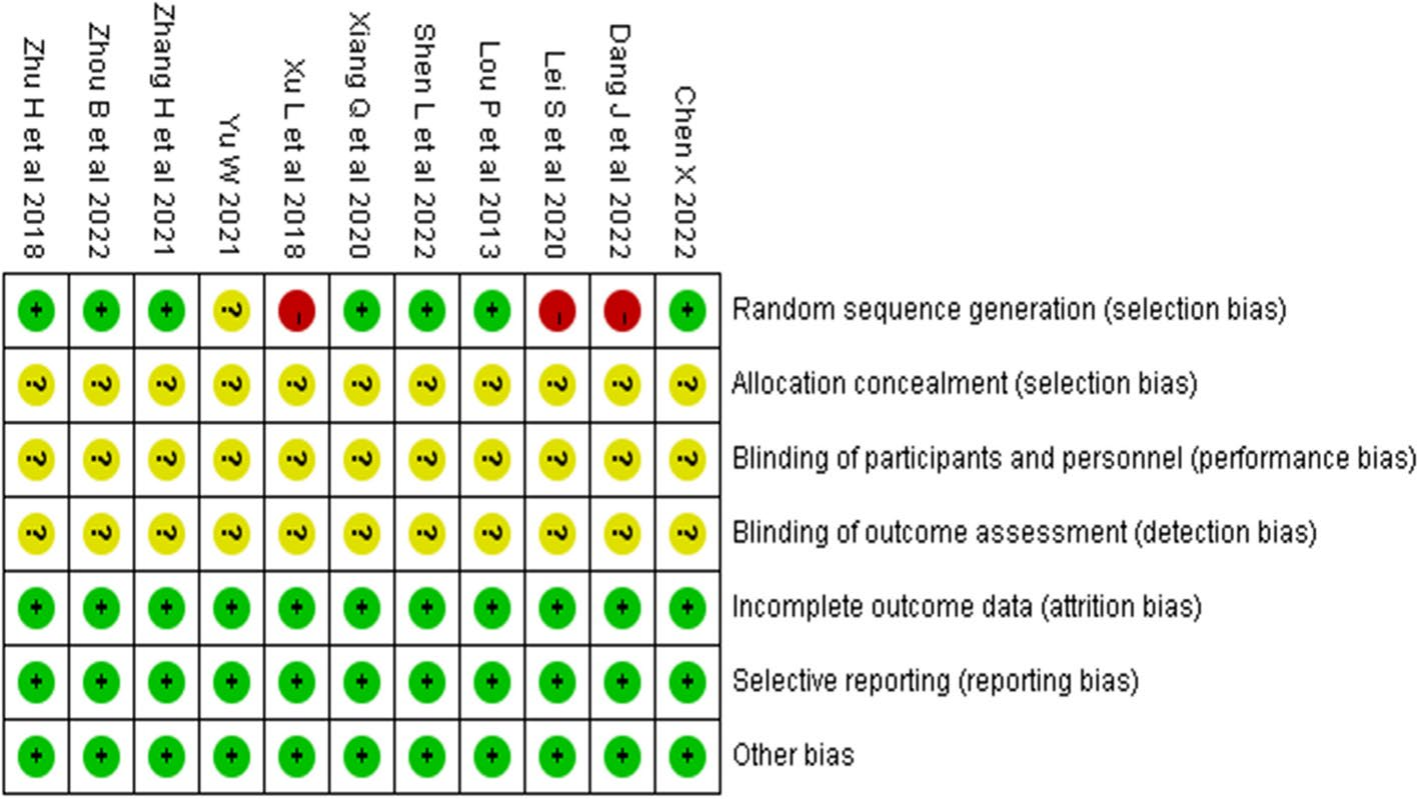
Risk of bias summary

Two researchers evaluated and graded the 11 included studies according to the RTC bias risk assessment tool [26] provided by the Cochrane Collaboration. The results are shown in Table 2 and Fig. 2. All studies were graded B in quality. Ten studies [27–32, 34–37] described the generation of randomized sequences, with seven studies [27–30, 32, 35, 37] using random number tables for grouping, one study [31] using odd-even numbering for grouping, one study [34] grouping according to patient preference, and one study [36] mentioning randomization but not specifying the method used. None of the 11 studies had any dropouts or missing data reports, and the experimental and control groups were comparable in terms of baseline levels before the intervention (*P* > 0.05). This suggests that the methodological quality of the included literature is fair, the risk of bias is low, and the credibility of the evidence is high.

**Table 2:** Risk of bias summary

Inclusion study	Random sequence generation	Assign hidden	Blinding of participants and personnel	Blinding of outcome assessment	Incomplete outcome data	Selective reporting	Other biases	Quality grade
Xiang Q et al 2020 [27]	Low	Unclear	Unclear	Unclear	Low	Low	Low	B
Zhou B et al 2022 [28]	Low	Unclear	Unclear	Unclear	Low	Low	Low	B
Shen L et al 2022 [29]	Low	Unclear	Unclear	Unclear	Low	Low	Low	B
Zhang H et al 2021 [30]	Low	Unclear	Unclear	Unclear	Low	Low	Low	B
Dang J et al 2022 [31]	High	Unclear	Unclear	Unclear	Low	Low	Low	B
Chen X 2022 [32]	Low	Unclear	Unclear	Unclear	Low	Low	Low	B
Yu W et al 2021 [33]	Unclear	Unclear	Unclear	Unclear	Low	Low	Low	B
Xu L et al 2018 [34]	High	Unclear	Unclear	Unclear	Low	Low	Low	B
Zhu H et al 2018 [35]	Low	Unclear	Unclear	Unclear	Low	Low	Low	B
Lei S et al 2020 [36]	Low	Unclear	Unclear	Unclear	Low	Low	Low	B
Lou P et al 2013 [37]	Low	Unclear	Unclear	Unclear	Low	Low	Low	B

Quality grade: B is medium quality

### Meta-analysis results and sensitivity analysis

#### Smoking cessation rates

**Fig. 3:**
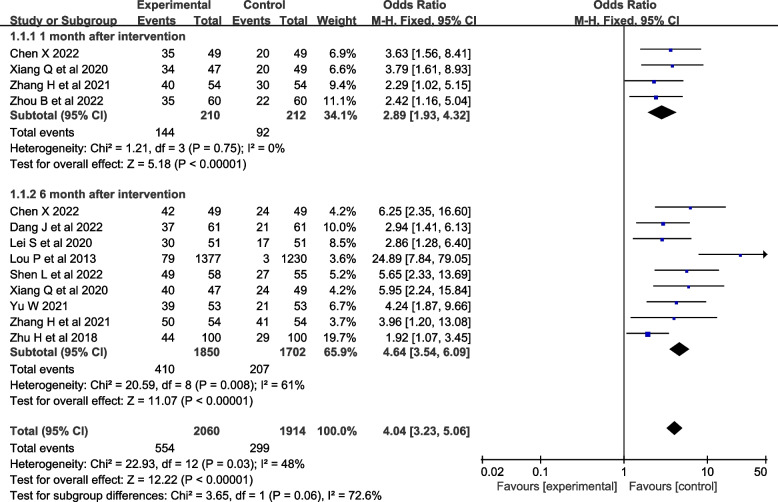
Forest plot of smoking cessation rate

Ten studies [27–29, 33, 35–37] were evaluated for smoking cessation rates. Four studies [27, 28, 30, 32] reported smoking cessation rates at one month after the intervention, and nine studies [27, 29–33, 35–37] reported smoking cessation rates at six months after the intervention. Fewer studies reported smoking cessation rates at three and twelve months after the intervention, so they were not included in the meta-analysis. The heterogeneity test was conducted, *I*^*2*^=48% and *P*=0.03, and the heterogeneity was acceptable. A fix-effects model was used for analysis, which showed that smoking cessation interventions at different intervention times were more effective in increasing smoking cessation rates than the control group [*OR*=4.04, *95%CI* (3.23, 5.06), *P*<0.001, Fig. 3].

#### Nicotine dependence

**Fig. 4:**
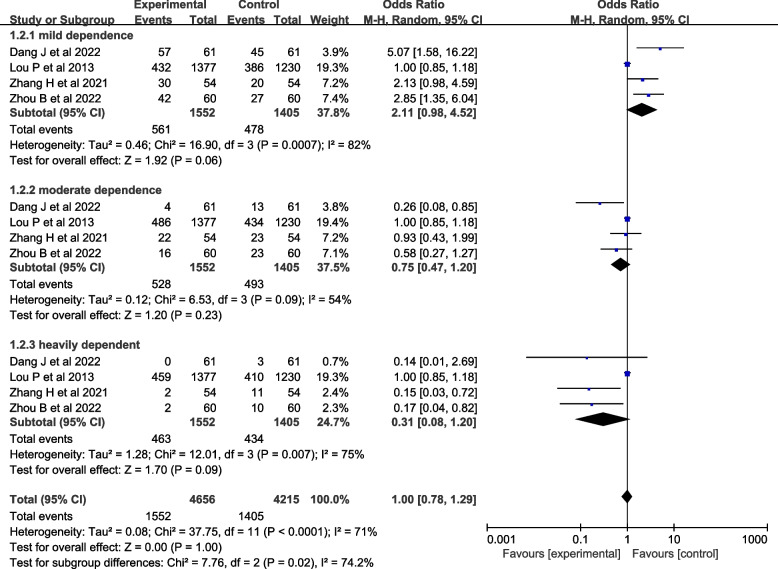
Forest plot of nicotine dependence level

Seven studies [27–32, 37] evaluated nicotine dependence. However, one study [27] used percentile and interquartile range to describe nicotine dependence, two studies [29, 32] used percentile and interquartile range to describe nicotine dependence, and four studies [28, 30, 31, 37] described nicotine dependence as mild, moderate, and severe, so four studies [28, 30, 31, 37] were included in the meta-analysis. A heterogeneity test was conducted, resulting in an *I*^*2*^=71% and *P*<0.001. A random-effects model was used for analysis, which showed that the effect of theory-based smoking cessation interventions on nicotine dependence could not be determined [*OR*=1.00, *95%CI* (0.78, 1.29), *P*<0.001, Fig. 4]. Sensitivity analysis was performed by excluding individual studies, and the results still showed significant heterogeneity, indicating that the heterogeneity was stable.

#### Pulmonary function

**Fig. 5:**
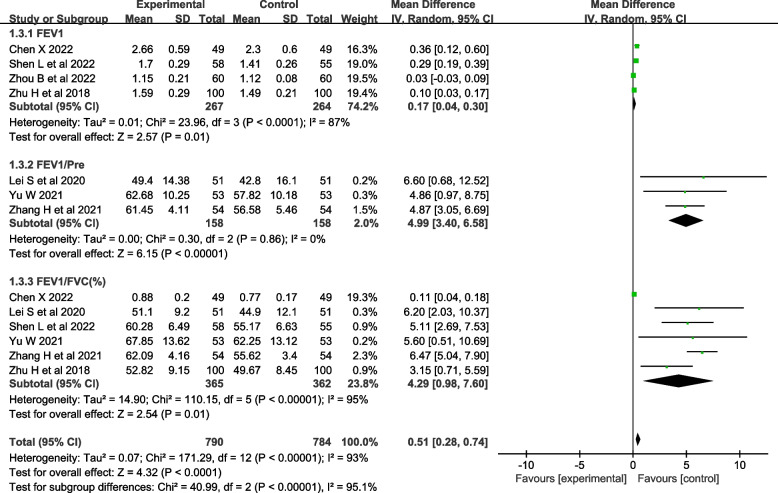
Forest plot of lung function

Seven studies [28–30, 32, 33, 35, 36] evaluated lung function, including FEV1 (forced expiratory volume in the first second) [28, 29, 32–35], FEV1/Pre (ratio of forced expiratory volume in the first second to estimated vital capacity) [30, 32–36], and FEV1/FVC (ratio of forced expiratory volume in the first second to forced vital capacity) [28, 30, 32, 33, 35, 36]. The heterogeneity test showed that there was significant heterogeneity in FEV1 and FEV1/FVC among the studies (*I*^*2*^>50%, *P*<0.001), and there was no heterogeneity in FEV1/Pre (*I*^*2*^=0%, *P*=0.86). A random-effects model was used for analysis, which showed that the effect of theory-based smoking cessation interventions on lung function was better in the experimental group than in the control group [*MD*=0.51, *95% CI* (0.28, 0.74), *P*<0.001, Fig. 5]. Sensitivity analysis was performed by excluding individual studies, and the results still showed significant heterogeneity, indicating that the heterogeneity was stable.

#### Quality of life

**Fig. 6:**
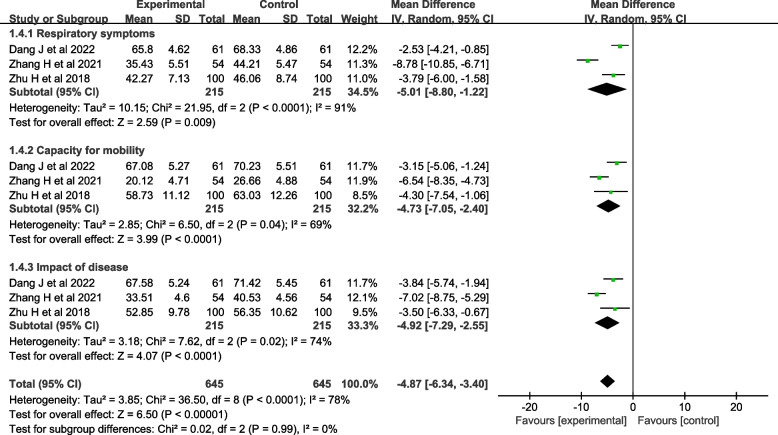
Forest plot of quality of life

Four studies [29–31, 35] evaluated quality of life. One study [29] used the Seattle COPD questionnaire [39] for evaluation, and three studies [30, 31, 35] used the St. George's Respiratory Questionnaire (SGRQ) [40] for evaluation, so three studies [30, 31, 35] were included in the meta-analysis. The heterogeneity test showed that there was significant heterogeneity (*I*^*2*^=78%, *P*<0.001). A random-effects model was used for analysis, which showed that the effect of theory-based smoking cessation interventions on quality of life was better in the experimental group than in the control group [*MD*=-4.87, *95% CI* (-6.34, -3.40), *P*< 0.001, Fig. 6]. Sensitivity analysis was performed by excluding individual studies, and the results still showed significant heterogeneity, indicating that the heterogeneity was stable.

#### Clinical symptom score

Two studies [28, 34] reported clinical symptom scores, which are not suitable for meta-analysis because of the paucity of literature. Both studies [28, 34] showed that the clinical composite symptom scores were significantly lower in the experimental group than in the control group (*P* <0.05).

#### Frequency of clinical symptom exacerbation

Two studies [33, 34] reported the frequency of clinical symptom exacerbation, which was not suitable for meta-analysis due to the small number of studies. The two studies [33, 34] both showed that the frequency of clinical symptom aggravation in the experimental group was significantly lower than that in the control group (*P*<0.05).

## Discussion

This study conducted a meta-analysis of data from 11 randomized controlled trials to assess the effectiveness of smoking cessation interventions in patients with COPD. This meta-analysis demonstrated that based on timing theory [10], 5A nursing model [12], and cognitive behavioral theory [13] smoking cessation interventions significantly improved smoking cessation rates, lung function, and quality of life in COPD patients. However, these interventions did not significantly affect nicotine dependence levels.

The timing theory proposes that smoking cessation strategies should be targeted based on the disease stage of COPD patients. This approach emphasizes understanding the different stages of the disease, improving negative behaviors, and increasing patients' confidence to quit smoking [27–33]. The 5A nursing model involves individualized assessment, setting goals, and providing help and regular follow-up to change COPD patients' cognition of the disease and the harm of smoking so that they can establish correct health beliefs [34, 35]. Cognitive behavioral theory emphasizes the importance of addressing patients' smoking-related thoughts and behaviors for successful smoking cessation [36, 37]. Healthcare providers can develop interventions by targeting the specific needs of patients at each stage of the disease, identifying the underlying causes of their smoking behavior, and selecting an appropriate rationale. The goal is to help COPD patients develop effective strategies to quit smoking and manage their disease symptoms. This study provides valuable insights into the effectiveness of theory-based smoking cessation interventions for COPD patients.

### Theory-based smoking cessation interventions can improve the smoking cessation rate of COPD patients

The findings of this study suggest that theory-based smoking cessation interventions can improve smoking cessation rates in patients with COPD. Given the strong association between COPD and smoking, it is crucial to address smoking cessation as a key component of COPD management [41]. Previous studies mainly used smoking cessation drugs to relieve withdrawal symptoms or used auxiliary methods to improve the success rate of smokers who wanted to quit, but not all patients were willing to accept or needed to use smoking cessation drugs to quit successfully [42, 41–44]. The positive impact of theory-based smoking cessation interventions on smoking cessation rates can be attributed to their emphasis on understanding patients' individual needs, motivations, and barriers to quitting smoking, as well as providing tailored support and strategies to overcome these challenges. By addressing the psychological aspects of smoking behavior and incorporating behavioral change theories, these interventions can help patients develop the necessary skills and confidence to successfully quit smoking. The use of theory-based interventions is particularly promising because it allows for a more systematic and evidence-based approach to smoking cessation. It is more conducive for patients to form a strong desire to quit smoking and take action to bring about more effective and sustainable smoking cessation effects for patients. The sensitivity analysis showed that the heterogeneity among the studies included in the meta-analysis was acceptable, indicating that the evidence results were relatively reliable.

### The effect of theory-based smoking cessation interventions on nicotine dependence levels is uncertain

Nicotine dependence, also known as tobacco dependence, is a chronic disease [45]. A considerable number of COPD patients, know the harm of smoking and have the intention to quit, but because they are addicted to smoking, it is difficult to quit, which means that their degree of tobacco dependence has not improved and they still have a high risk of relapse after discharge [46]. The lack of significant effect on nicotine dependence levels may be due to several factors, including the relatively short duration of the interventions and follow-up periods in the included studies, as well as potential differences in the measurement and reporting of nicotine dependence levels across studies. For patients, in addition to providing professional and scientific help throughout the smoking cessation process, better results can be achieved by combining drug control and encouraging family members to provide adequate emotional support throughout the process. It is recommended that future studies be guided by theory and combined with pharmacological control to investigate the improvement effect.

### Theory-based smoking cessation interventions improve lung function and quality of life in COPD patients

Lung function is the gold standard for diagnosing and evaluating the severity of COPD, which can objectively reflect the degree of airflow restriction or obstruction in patients [47]. Due to the intake of a large amount of nicotine, tar, and some radioactive substances, COPD smokers have a serious impact on their lung health, which not only causes inflammatory changes but also threatens the lung function of the body's respiratory system [48]. As the duration of smoking increases, the lung function of patients also decreases, which further triggers a series of lung diseases and reduces their quality of life [49, 50], so it is urgent to control their smoking behavior.

The improvement in lung function observed in this meta-analysis is consistent with previous research showing that smoking cessation can lead to significant improvements in lung function and reduce the risk of COPD exacerbations. By helping patients quit smoking, theory-based interventions may contribute to slowing down the progression of COPD and improving patients' overall respiratory health. The observed improvement in quality of life is also an important finding, as COPD is known to have a significant impact on patients' physical, emotional, and social well-being. By addressing both the physical and psychological aspects of smoking behavior, theory-based interventions may help improve patient’s overall well-being and quality of life.

### Limitations

Several limitations of this study remain: (i) Due to language limitations, only publicly available Chinese and English literature was searched, which may result in incomplete literature collection; (ii) The included studies did not mention allocation concealment and blinding methods, resulting in medium-quality research quality, which may affect the reliability of the results to some extent. It is hoped that subsequent relevant research will further improve the rigor of allocation concealment and blinding methods to achieve higher quality levels. (iii) Currently, most studies only report short-term effects of theory-based smoking cessation interventions on COPD patients.

## Conclusion

The findings of this study demonstrated that implementing theory-based smoking cessation interventions in conventional healthcare can have a positive effect on the smoking cessation rate, lung function, and quality of life of COPD patients. It is recommended that these interventions be widely implemented in clinical practice. Further investigation is required to confirm these findings due to the limitations in the standardization and homogeneity of the included studies.

### Supplementary Information

**Additional file 1.** Search terms and strategies.

**Additional file 2.** PRISMA Checklist.

## Data Availability

The study is conducted using open-source data from published articles. Additional data can be made available upon request to Mengjing Han(hmjgz986@163.com).
