# Supplementary material for: The effectiveness of theory-based smoking cessation interventions in patients with chronic obstructive pulmonary disease: a meta-analysis
Source: BMC Public Health. 2023 Aug 9;23:1510. doi: 10.1186/s12889-023-16441-w (PMC10410903; doi:10.1186/s12889-023-16441-w)
Supplement: Supplementary file 1 — Additional file 1. Search terms and strategies. [file 12889_2023_16441_MOESM1_ESM.docx]

**Search terms and strategies**

Full search terms and strategies in **Chinese electronic databases** including CNKI, Wanfang Knowledge Service platform, VIP resource integration service platform, and China Biomedical Literature Database

#1 (Chronic Obstructive Pulmonary Disease OR COPD OR Chronic Obstructive Airway Disease OR Airflow Obstruction, Chronic OR Chronic Airflow Obstruction)

#2 (Smoking cessation OR Smoking cessation intervention)

#3 (theory OR theoretical OR model OR theoretical model)

#1 AND #2 AND #3

No restrictions were set on literature type or publication status.

**PubMed**

# Search: ("chronic obstructive lung disease*"[Title/Abstract] OR "chronic obstructive pulmonary disease*"[Title/Abstract] OR "chronic obstructive airway disease*"[Title/Abstract] OR "COAD"[Title/Abstract] OR "COPD"[Title/Abstract] OR "airflow obstruction* chronic"[Title/Abstract] OR "chronic airflow obstruction*"[Title/Abstract] OR "Pulmonary Disease, Chronic Obstructive"[Title/Abstract]) AND ("theory"[Title/Abstract] OR "Theoretical"[Title/Abstract] OR "model*"[Title/Abstract] OR "theoretical model*"[Title/Abstract] OR "model* theoretical"[Title/Abstract] OR "mathematical model*"[Title/Abstract] OR "model* mathematical"[Title/Abstract] OR "theoretical stud*"[Title/Abstract] OR ("stud* theoretical"[Title/Abstract]) AND "cessation smoking"[Title/Abstract] OR "smoking quitting"[Title/Abstract] OR "smoking cessations"[Title/Abstract] OR "stopping smoking"[Title/Abstract] OR "smoking stopping"[Title/Abstract] OR "giving up smoking"[Title/Abstract] OR "smoking giving up"[Title/Abstract] OR "quitting smoking"[Title/Abstract]

**Web of Science**

You searched for : (TS= (chronic obstructive lung disease*OR chronic obstructive pulmonary disease* OR chronic obstructive airway disease* OR COAD OR COPD OR airflow obstruction* chronic OR chronic airflow obstruction* OR Pulmonary Disease, Chronic Obstructive)) AND (TS= (theory OR theoretical OR model* OR theoretical model* OR model* theoretical OR mathematical model* OR model* mathematical OR theoretical stud* OR stud* theoretical)) AND (TS= (cessation smoking OR smoking quitting OR smoking cessations OR stopping smoking OR smoking stopping OR giving up smoking OR smoking giving up OR quitting smoking))

**Embase**

1 chronic obstructive lung disease*':ab,ti

2 chronic obstructive pulmonary disease*':ab,ti

3 chronic obstructive airway disease*':ab,ti

4 COAD':ab,ti

5 COPD':ab,ti

6 airflow obstruction* chronic':ab,ti

7 chronic airflow obstruction*':ab,ti

8 Pulmonary Disease, Chronic Obstructive':ab,ti

9 1 or 2 or 3 or 4 or 5 or 6 or 7 or 8

10 theory':ab,ti

11 theoretical':ab,ti

12 model*':ab,ti

13 theoretical model*':ab,ti

14 model* theoretical':ab,ti

15 mathematical model*':ab,ti

16 model* mathematical':ab,ti

17 theoretical stud*':ab,ti

18 stud* theoretical':ab,ti

19 10 or 11 or 12 or 13 or 14 or 15 or 16 or 17 or 18

20 cessation smoking':ab,ti

21 smoking quitting':ab,ti

22 smoking cessations

23 stopping smoking':ab,ti

24 smoking stopping':ab,ti

25 giving up smoking':ab,ti

26 smoking giving up':ab,ti

27 quitting smoking':ab,ti

28 20 or 21 or 22 or 23 or 24 or 25 or 26 or 27

29 9 and 19 and 28

**The Cochrane Library**

#1 Mesh descriptor: [Pulmonary disease. chronic obstructive] explode all trees

#2 (chronic obstructive lung disease*OR chronic obstructive pulmonary disease* OR chronic obstructive airway disease* OR COAD OR COPD OR airflow obstruction* chronic OR chronic airflow obstruction* OR Pulmonary Disease, Chronic Obstructive)

#3 #1 or #2

#4 Mesh descriptor: [theoretical] explode all trees

#5 (theory OR theoretical OR model* OR theoretical model* OR model* theoretical OR mathematical model* OR model* mathematical OR theoretical stud* OR stud* theoretical)

#6 #4 or #5

#7 Mesh descriptor: [smoking cessation] explode all trees

#8 (cessation smoking OR smoking quitting OR smoking cessations OR stopping smoking OR smoking stopping OR giving up smoking OR smoking giving up OR quitting smoking)

#9 #7 or #8

#10 #3 and #6 and #9

**Table 1 Basic characteristics of the included literature**

| Inclusion study | Sample | Age | | Intervention methods | | Outcome indicators |
| --- | --- | --- | --- | --- | --- | --- |
|  | T/C | T | C | T | C |  |
| Xiang Q et al 2020 | 47/49 | 62.75±11.34 | 64.52±13.94 | the timing theory | routine nursing care | ①② |
| Zhou B et al 2022 | 60/60 | 62.89±2.87 | 63.10±4.12 | the timing theory | routine nursing care | ①②③⑤ |
| Shen L et al 2022 | 58/55 | 63.39±9.28 | 63.08±9.45 | the timing theory | routine nursing care | ①②③④ |
| Zhang H et al 2021 | 54/54 | 53.90±6.31 | 53.26±6.53 | the timing theory | routine nursing care | ①②③④ |
| Dang J et al 2022 | 61/61 | 60.42±2.75 | 60.18±2.69 | the timing theory | routine nursing care | ①②④ |
| Chen X 2022 | 49/49 | 62.76±11.35 | 64.49±13.96 | the timing theory | routine nursing care | ①②③ |
| Yu W et al 2021 | 53/53 | 67.83±9.62 | 68.25±9.75 | the timing theory | routine nursing care | ①③⑥ |
| Xu L et al 2018 | 79/79 | 57.53±3.04 | 55.94 ± 2.67 | the 5A nursing model | routine nursing care | ⑤⑥ |
| Zhu H et al 2018 | 100/100 | 58.81±11.49 | 59.73±11.64 | the 5A nursing model | routine nursing care | ①③④ |
| Lei S et al 2020 | 51/51 | 61.10±12.30 | 61.50±12.50 | the cognitive behavioral theory | routine nursing care | ①③ |
| Lou P et al 2013 | 1377/1230 | 61.60±10.20 | 61.50±10.10 | the cognitive behavioral theory | routine nursing care | ①② |

Note: T: test group, C: control group; ①quit rate; ②nicotine dependence; ③lung function; ④quality of life; ⑤clinical symptom score; ⑥frequency of clinical symptom exacerbation

**Table 2 Risk of bias summary**

| Inclusion study | Random sequence generation | Assign hidden | Blinding of participants and personnel | Blinding of outcome assessment | Incomplete outcome data | Selective reporting | Other biases | Quality grade |
| --- | --- | --- | --- | --- | --- | --- | --- | --- |
| Xiang Q et al 2020 | Low | Unclear | Unclear | Unclear | Low | Low | Low | B |
| Zhou B et al 2022 | Low | Unclear | Unclear | Unclear | Low | Low | Low | B |
| Shen L et al 2022 | Low | Unclear | Unclear | Unclear | Low | Low | Low | B |
| Zhang H et al 2021 | Low | Unclear | Unclear | Unclear | Low | Low | Low | B |
| Dang J et al 2022 | High | Unclear | Unclear | Unclear | Low | Low | Low | B |
| Chen X 2022 | Low | Unclear | Unclear | Unclear | Low | Low | Low | B |
| Yu W et al 2021 | Unclear | Unclear | Unclear | Unclear | Low | Low | Low | B |
| Xu L et al 2018 | High | Unclear | Unclear | Unclear | Low | Low | Low | B |
| Zhu H et al 2018 | Low | Unclear | Unclear | Unclear | Low | Low | Low | B |
| Lei S et al 2020 | Low | Unclear | Unclear | Unclear | Low | Low | Low | B |
| Lou P et al 2013 | Low | Unclear | Unclear | Unclear | Low | Low | Low | B |

Note: Quality grade: B is medium quality.


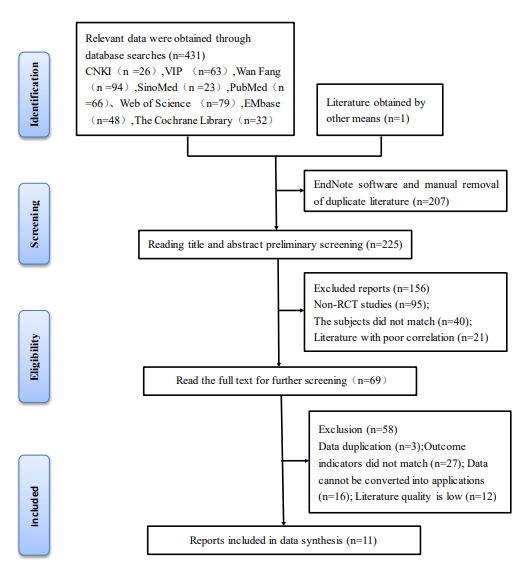


**Figure 1 Flow chart of literature screening**


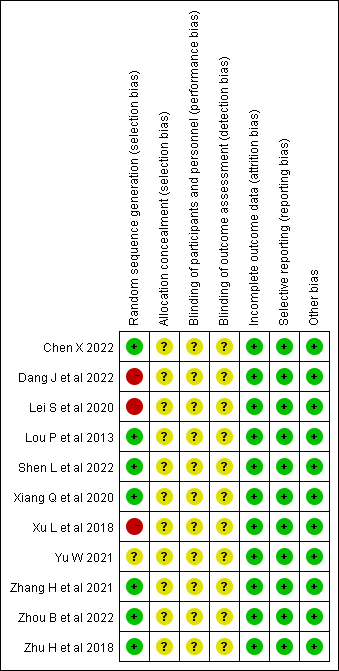


**Figure 2 Risk of bias summary**


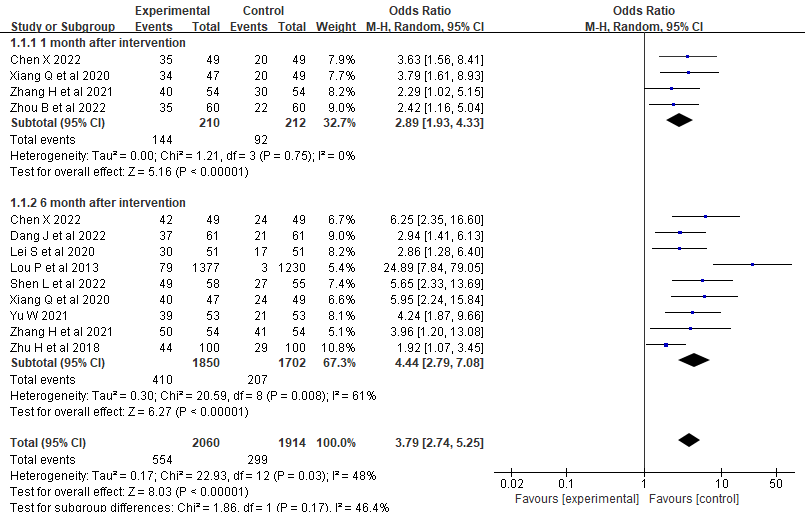


**Figure 3 Forest plot of smoking cessation rate**


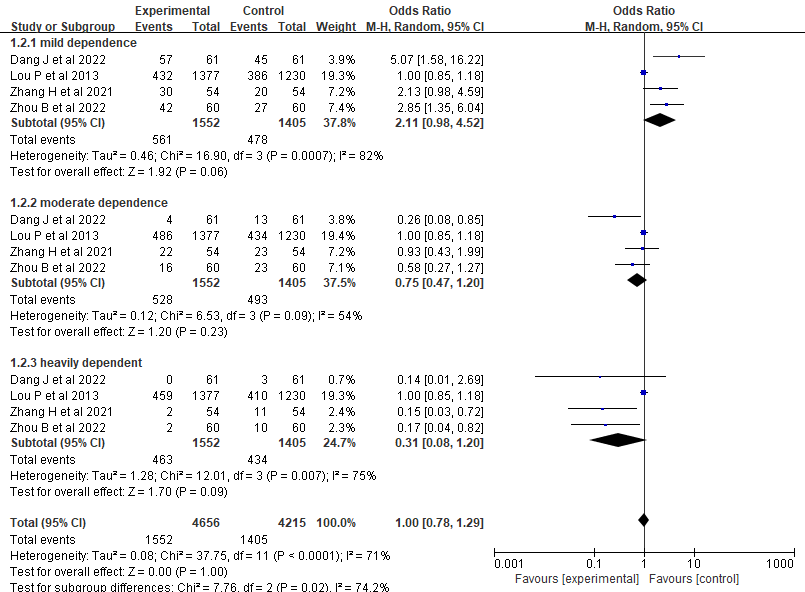


**Figure 4 Forest plot of nicotine dependence level**


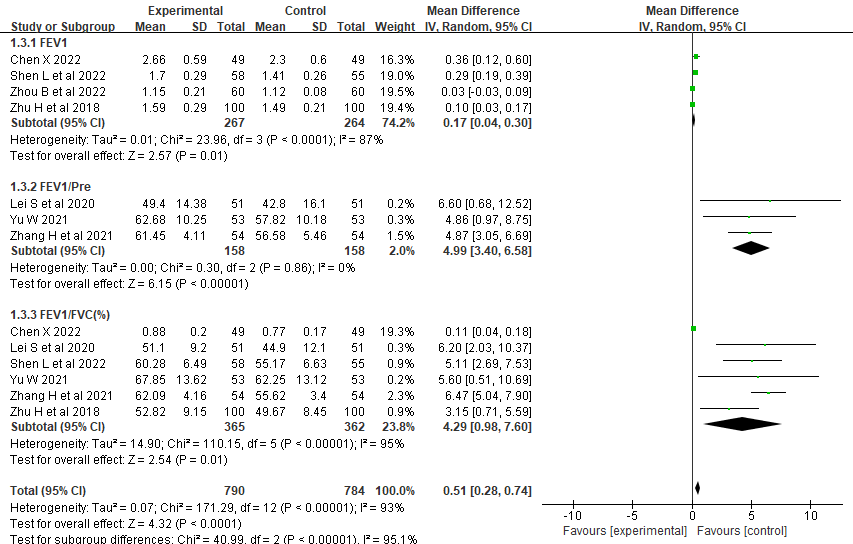


**Figure 5 Forest plot of lung function**

**
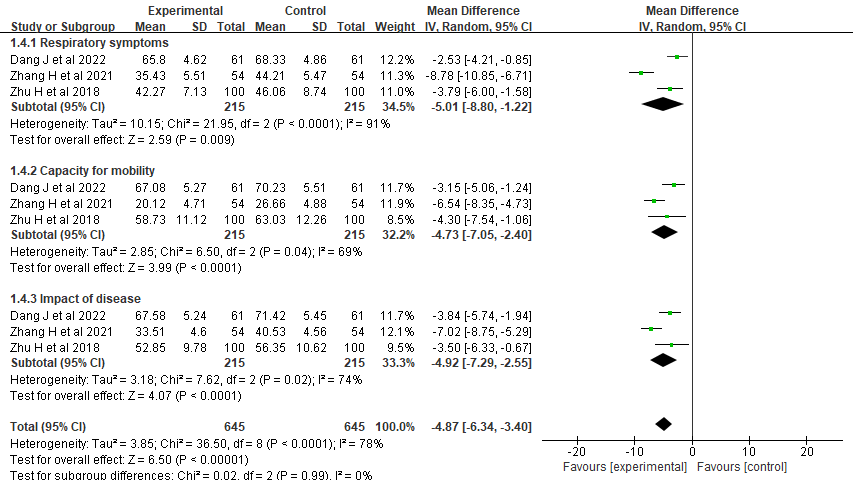
**

**Figure 6 Forest plot of quality of life**
